# Higher PAPP-A Values in Pregnant Women Complicated with Preeclampsia Than with Gestational Hypertension

**DOI:** 10.1007/s43032-023-01176-1

**Published:** 2023-03-14

**Authors:** Montserrat Uriel, Ximena Carolina Romero Infante, Sara Rincón Franco, Edgar Antonio Ibáñez Pinilla, Nydia Alexandra Rojas

**Affiliations:** 1 El Bosque Research Group of Maternal Fetal Medicine and Gynecology, Universidad El Bosque, Bogotá, Colombia; 2 Ecodiagnóstico El Bosque S.A.S., Bogotá, Colombia; 3 Los Cobos Medical Center, Bogotá, Colombia; 4 Research Group of Community Medicine and Collective Health, Universidad El Bosque, Bogotá, Colombia

**Keywords:** Pregnancy-associated Plasma Protein-A (PAPP-a), Preeclampsia, IUGR, Gestational Hypertension, Hypertensive Disorders of Pregnancy

## Abstract

The purpose of this study is to compare the levels of maternal serum pregnancy–associated plasma protein-A at the first trimester in pregnancies complicated by impaired placental diseases, such as preeclampsia (PE), intrauterine fetal growth restriction (IUGR), and gestational hypertension (GH), with those in pregnancies without the development of any of these outcomes to expand the knowledge of how this protein behaves in the different impaired placental diseases. This current work is an observational study based on a prospective cohort. Pregnancy-associated plasma protein-A was measured in 422 patients who had completed maternal-perinatal outcomes. Comparisons of pregnancy characteristics and the biomarker between outcome groups (PE, IUGR, gestational hypertension, and not impaired placental outcomes) were analyzed. PAPP-A MoM in the IUGR (0.8 IQR: 0.6–0.9) and GH groups (0.5 IQR: 0.3–1.4) compared to the PE group (1.06 IQR: 0.66–1.52) was significantly lower (*p* < 0.005). Pregnant women who developed early-onset PE (1.11 IQR 1.08–1.18) presented significant differences with the IUGR group (0.83 IQR: 0.59–0.98; *p* = 0.002) and those who developed preterm-PE (1.19 IQR: 0.66–1.58; *p* = 0.045). The results demonstrate that the levels of PAPP-A at first trimester in the sample of women who developed PE, and specially term-PE, were higher than those in women who developed GH or IUGR. The GH group had the lowest PAPP-A values in this sample of pregnant women. Research in a population with a high prevalence of preeclampsia is still lacking and deserves more extended studies to define if these patients could have different rates of PAPP-A.

## Introduction

Health in obstetric involves two patients: mother and fetus. For decades, the World Health Organization (WHO) has been working to reduce morbidity and mortality in obstetrics, improving quality prenatal care and introducing strategies to decrease the severity of obstetric diseases. One of the most dangerous illnesses during pregnancy is PE. PE is an obstetric pathology that is classified in the group of illnesses that have an inappropriate process of placentation. Consequently, these women suffer from different diseases related to vasoconstriction and hypertensive disorders and from gestational hypertension to PE, and it can also affect the fetus, which receives fewer amounts of nutrients and oxygen during pregnancy, reducing its potential growth [1–5].

Hypertensive disorders of pregnancy affect 5–8% of pregnant women worldwide each year and are among the obstetric diseases with the worst gestational outcomes, with high maternal-perinatal morbidity and mortality [6–10], secondary to maternal and fetal complications such as IUGR (42). The prevalence of PE is around 2–4% worldwide [4, 11]; however, Colombia has a prevalence of PE of 6–8%, and it was the first or second cause of maternal mortality in the last years [5, 12, 13]. The prevalence of gestational hypertension (GH), also among hypertensive disorders of pregnancy, is around 2–4%, and its relationship with biomarkers has been less studied [14, 15].

The similar physiopathology of these diseases is the cause of which prediction models for PE, GH, and IUGR involve the same biomarker at first trimester of pregnancy, such as the maternal serum pregnancy-associated plasma protein-A (PAPP-A). This biomarker is produced by the placental tissue, and the improper trophoblastic invasion in pregnant women, affected by impaired placental diseases, generates a diminution of its levels, due to the decrease in placental function [14–19]. PAPP-A began to be used in biochemical screening for trisomies 21, 18, and 13. Because of its relation to placental function, it became a target of study about screening for PE and IUGR. These trials could determine that low-serum levels of PAPP-A were associated with a higher incidence of PE and IUGR. Since this discovery, PAPP-A biomarker is part of the model of early screening for PE and IUGR at first trimester of pregnancy that is used nowadays [7–9, 18, 20–23].

The precise early counselling, applying prediction models, could decrease maternal and fetal morbidity and mortality through pharmacological prevention by a low dose of acetylsalicylic acid (ASA) beginning before fourteen gestational weeks [24]. These models that combined maternal demographic characteristics and biophysical and biochemical markers of impaired placentation can identify a high proportion of pregnancies at risk of PE and/or IUGR [25–30]. However, one of the limitations of these studies, which were used to obtain the screening combined test, have been made in populations with lower incidence of PE than in other countries, such as Colombia [4].

This study aims to compare the levels of PAPP-A at first trimester in pregnancies complicated with PE, IUGR, and GH and of those without the development of any impaired disease.

## Materials and Methods

### Study Population

This is an observational study based on a prospective cohort of an ongoing research study about diagnostic validation of hypertensive disorders’ biomarkers in Colombian pregnant women. A total of 566 patients were included between November 2013 and August 2017, and three main institutions took part in this multicenter study, Ecodiagnóstico El Bosque Diagnostic Unit Centre, El Bosque Clinic, and South Kennedy Hospital (including West Health Services Unit) at Bogotá (Colombia). The population of interest was single pregnancies who had completed maternal-perinatal outcomes, and the exclusion criteria were threatened abortion, major fetal anomalies, fetal chromosomopathies, and maternal age below 14 years old.

Maternal medical history was taken at the inclusion time with a special request of different hypertension risk factors, and, for physical examination, maternal blood pressure and weight were measured. In addition, pregnancy-associated plasma protein-A (PAPP-A) was taken at the same time of the first-trimester scan for chromosomal abnormalities.

Maternal and fetal outcomes were collected by clinical records to group patients according to the presented impaired placenta illness at the delivery.

### Maternal History

Searched maternal history included maternal age, racial origin (Caucasian, Afro-Caribbean, Asian, and mixed), economic level, smoking habit, method of conception, personal chronic pathologies (hypertension, systematics lupus erythematosus, renal pathology, antiphospholipid syndrome, and diabetes mellitus), and also personal and family history of PE and IUGR. Also, parity was asked, and, if it was not the first pregnancy, the new paternity was investigated and registered. Regarding the early preventive measures such as ASA intake, this is a blind study without any type of medical intervention by the research, so this was not considered.

Weight, height, and mean arterial pressure (MAP) were measured by calibrated equipment. Blood pressure was taken by automated devices (Microlife, BP A100 Plus, Taipéi, Taiwán) twice in both arms to calculate MAP.

Maternal serum biomarkers were measured using the DELFIA XPRESS (PerkinElmer, Inc.). To standardize according to maternal and pregnancy characteristics, every biomarker’s value was converted to a multiple of the expected normal median (MoM) specific to a pregnancy of the same gestational age, maternal weight, racial origin, smoking habit, method of conception, and parity as it’s described in the previous scientific literature [17, 19, 26].

### Outcome Measures

Obstetric records collected by clinical history were examined to establish four groups according to impaired placentation disease (PE, IUGR, and GH) and patients without the development of these outcomes. Posteriorly, in order to know if there would be different results if PE disease appears before or after 34 weeks and before or after 37 weeks, a new analysis was done with five groups. Firstly, patients were grouped in gestations without the presence of impaired placentation disease or normal, PE after 37 weeks (term PE), PE before 37 weeks (preterm PE), IUGR, and GH. Lastly, patients were grouped in normal gestations, PE after 34 weeks (late-onset PE), PE before 34 weeks (early-onset PE), IUGR, and GH [15].

The definitions of PE and gestational hypertension were taken from the last Task Force on Hypertension in Pregnancy [31, 32]: the systolic blood pressure should be 140 mm Hg or more, and/or the diastolic blood pressure should be 90 mm Hg or more, on at least two occasions 4 h apart, after 20 weeks of gestation in previously normotensive women. In the case of PE, pregnant women should have proteinuria of 300 mg or more in 24 h or present any criteria of severe PE in addition.

The criteria to establish IUGR diagnosis was a fetal or neonatal weight below the third percentile for gestational age or below the 10th percentile and alteration of fetoplacental circulation [33, 34].

Women who had a diagnosis of PE and IUGR were included in the group of PE because in both cases, the cause of finalization of pregnancy was PE.

### Ethical Considerations

El Bosque University Ethical Committee agreed with this study, so the written informed consent was signed by every pregnant woman who participated in the study. In addition, the ethical principles for human research from the Helsinki Declaration and the Colombian Resolution 8430 of 1993 were considered in this study [35, 36]. The privacy of each patient was respected throughout the study.

### Statistical Analysis

Comparisons of pregnancy characteristics and the biomarker between outcome groups were analyzed, conducted both in mean and median, by the Student’s *t* test for variables with Gaussian distribution, Mann–Whitney *U* test for variables without Gaussian distribution, and square Ji Pearson’s test for categorical variables (adjusted significance level *p* < 0.05 and *p* < 0.01). Shapiro–Wilk test (*p* > 0.05, normality) was used to determine Gaussian distribution.

The statistical software package SPSS 24.0 (IBM SPSS Statistics for Windows, Version 24.0; IBM Corp., Armonk, NY, USA) was used for all data analyses.

## Results

Pregnancy-associated plasma protein-A was analyzed in 422 single pregnancies. Of these, 360 patients had a normal pregnancy (without development of impaired placentation disease), 32 (7.6%, CI95% 5.3–10.41) developed PE (PE), 14 (3.3% CI95% 1.6–5.0) developed IUGR, and 16 (3.8% CI95% 2.0–5.6) developed GH.

The mean age was 27.4 ± 6.5 years old, and most of the patients were of mixed race (96.4%); only 7 pregnant women (1.7%) were Afro-American, and 8 (1.9%) were Caucasian. Most of the pregnant women had a low-middle socioeconomic level: 46.9% of patients had a low level and 48.3% middle.

A total of 156 (37%) were nulliparous, and 266 (63%) were multiparous; from this group of multiparous women, 37.5% (*n* = 100) had a new couple (new paternity). A proportion of 7.1% of women smoked previously or during pregnancy. At first trimester, the mean body mass index (BMI) was 24.6 ± 4 kg/m2, and only in 7 pregnant women (1.6%), the BMI was more than 35 kg/m2. The mean MAP at first trimester was within normal limits in every patient (80 ± 7 mmHg).

The percentage of pregnant women who had history of any chronic disease was 3.3%: 8 (1.9%) had chronic hypertension, 3 (0.7%) were diabetic, 1 (0.2%) had systematic erythematous lupus, 1 (0.2%) had antiphospholipid antibody syndrome, and 2 (0.5%) had chronic nephropathy. Regarding previous history of PE and IUGR, of these 266 multiparous, 31 (11.7%) had PE in previous pregnancies, and 15 (5.6%) had had a baby with IUGR previously. A total of 77 women (18.2%) had family history of PE (mother and/or sisters), and 29 (6.2%) had family history of IUGR.

The mean gestational age and the mean neonatal birth weight were 38 weeks plus 3 days ± 1 week plus 5 days and 3012.3 ± 464.9 g, respectively.

Table 1 shows the comparison of sociodemographic and clinical data between groups according to outcomes. No statistical differences were found (*p* > 0.05).

**Table 1:** Demographic characteristics of outcome groups

Characteristics	Statistics	No impaired placentation disease	Preeclampsia	Intrauterine growth restriction	Gestational hypertension
		(*n* = 360)	(*n* = 32)	(*n* = 16)	(*n* = 14)
Maternal age (years)	Mean (SD)	27.34 (6.5)	28.13 (6.96)	26.69 (6.36)	27.64 (7.55)
	Median (IQR)	27 (23–32)	29 (21–34)	26 (22–32)	27 (22–34)
Racial origin	Mixed, *n* (%)	345 (95.8)	32 (100)	16 (100)	14 (100)
Socioeconomic level	High, *n* (%)	18 (5)	2 (6.3)	0 (0)	0 (0)
	Low, *n* (%)	170 (47.2)	12 (37.5)	8 (50)	8 (57.1)
	Middle, *n* (%)	172 (47.8)	18 (56.3)	8 (50)	6 (42.9)
Smoking habit	Yes, *n* (%)	26 (7.2)	2 (6.3)	2 (12.5)	0 (0)
Chronic diseases	Yes, *n* (%)	9 (2.5)	4 (12.5)	1 (6.3)	0 (0)
Chronic Hypertension	Yes, *n* (%)	6 (1.6)	2 (6.2)	2 (12.5)	-
Familiar history of PE	Yes, *n* (%)	62 (17.2)	11 (34.4)	1(6.3)	3 (21.4)
Personal history of PE	Yes, *n* (%)	22 (6.1)	5 (15.6)	1 (6.3)	3 (21.4)
Familiar history IUGR	Yes, *n* (%)	22 (6.1)	6 (18.8)	1 (6.3)	0 (0)
Personal history of IUGR	Yes, *n* (%)	10 (2.8)	1 (3.1)	2 (12.5)	2 (14.3)
Parity	First pregnancy*, n* (%)	133 (36.9)	12 (37.5)	8 (50)	3 (21.4)
	Multiparous, new paternity, *n* (%)	84 (23.3)	9 (28.1)	4 (25)	3 (21.4)
	Multiparous, no new paternity, *n* (%)	143 (39.7)	11 (34.4)	4 (25)	8 (57.1)
Body mass index (BMI)	Mean (SD)	24.59 (3.93)	24.95 (4.96)	23.02 (2,75)	27.56 (5.16)
	Median (IQR)	24.1 (21.8–26.6)	23.69 (21.7–28.0)	22.9 (21–24)	26.9 (23.3–31.7)
Gestational age (weeks) at the moment of inclusion	Mean (SD)	12.75 (0.69	12.67 (0.67)	12.74 (0.77)	13.03 (0.48)
	Median (IQR)	12.6 (12.2–13.3)	12.6 (12.4–13.2)	12.9 (12.2–13.3)	13.1 (12.6–13.4)
Fetal CRL (mm) at the moment of inclusion	Mean (SD)	66.23 (9.46)	65.02 (8.46)	65.49 (9.60)	69.99 (7.86)
	Median (IQR)	65.6 (59–73.6)	66 (60.3–71.4)	66.1 (58.2–70.5)	69.9 (65–78)
Gestational age (weeks) at delivery	Mean (SD)	38.70 (1.54)	36.79 (2.45)	36.56 (2.49)	38.36 (1.10)
	Median (IQR)	39.00 (38.00–39.06)	37.00(36.0–39.0)	37.35 (36.42–37.57)	38.45 (37.5–39.02)
Mean neonatal birth weight (g)	Mean (SD)	3031.83 (423.98)	2619.97(689.47)	2073.76 (407.87)	3024.83 (366.71)
	Median (IQR)	3070.00 (2830–3320)	2860.0 (2395–3187.5﻿)	2242.5 (2056.25–2391.25)	3085.0 (2730–3251.25)

PE, preeclampsia; IUGR, intrauterine growth restriction; CRL, crown rump length

**Fig. 1:**
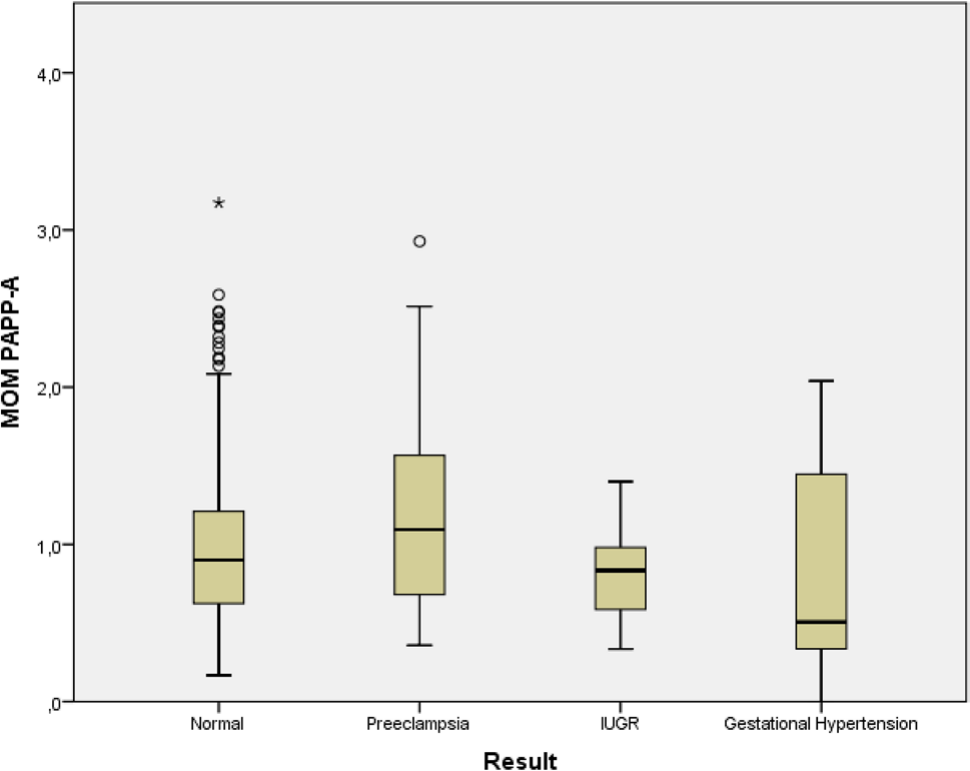
Box-and-whisker plots of multiple of median values (MOM) of PAPP-A. In the normal (no impared placentation disease), preeclampsia, IUGR (intrauterine growth restriction) and gestational hypertension groups

The distribution of levels of PAPP-A at first trimester in the studied patients are described in Fig. 1 and Table 2. Comparisons between groups were done. Firstly, (a) no impaired placentation outcome in contrast with those with a pathological outcome such as PE, IUGR, and pregnant women with GH. A second analysis (b) compared the pregnant women whose babies were diagnosed with IUGR with patients who developed PE or GH; and finally, a third analysis (c) was done to compare patients with GH with those pregnant women who developed PE. The results of the IUGR and GH groups (0.83 IQR: 0.59–0.98; 0.51 IQR: 0.34–1.45, respectively) were significantly lower compared to the PE group (1.06 IQR: 0.66–1.52); *p* = 0.041; *p* = 0.042, respectively. No other significant differences were found.

**Table 2:** PAPP-A of outcome groups

Characteristics	Statistics	No impaired placentation disease	Preeclampsia	Intrauterine growth restriction	Gestational hypertension
		(*n* = 360)	(*n* = 32)	(*n* = 16)	(*n* = 14)
MoM PAPP-A	Mean (SD)	1.00 (0.50)	1.15 (0.63)	0.79 (0.29)	0.79 (0.65)
	Median (IQR)	0.90 (0.63–1.21)	1.06 (0.66–1.52) (b*)(c*)	0.83 (0.59–0.98)	0.51 (0.34–1.45)

(a) Comparison with patients without impaired placentation disease. (b) Comparison with patients with development of intrauterine growth restriction. (c) Comparison with patients with gestational hypertension. There was no statistical significance with (a). *Significant at *p* < 0.05. **Significant at *p* < 0.01. SD, standard deviation; IQR, interquartile range

Different results were found when the PE group was divided into two groups, depending on presentation of the disease after o before the 34th week. Only the patients of the subgroup of pregnant women who developed early-onset PE (1.11 IQR 1.08–1.18) presented significant differences from the IUGR group (0.83 IQR: 0.59–0.98); *p* = 0.002 (Table 3).

**Table 3:** PAPP-A of outcome groups insolated PE into early-onset PE and late-onset PE ≥ 34w

Characteristics	Statistics	No impaired placentation disease	Early-onset PE, < 34w	Late-onset PE, ≥ 34w	Intrauterine growth restriction	Gestational hypertension
		(*n* = 360)	(*n* = 5)	(*n* = 27)	(*n* = 16)	(*n* = 14)
MoM PAPP-A	Mean (SD)	1.00 (0.50)	1.14 (0.11)	1.15 (0.68)	0.79 (0.29)	0.79 (0.65)
	Median (IQR)	0.90 (0.63–1.21)	1.11 (1.08–1.18) (b**)	0.92 (0.60–1.58)	0.83 (0.59–0.98)	0.51 (0.34–1.45)

(a) Comparison with normal, (b) comparison with intrauterine growth restriction, (c) comparison with gestational hypertension, (d) comparison with PE≥34w. There was no statistical significance with (a), (c), and (d). *Significant at *p* < 0.05. **Significant at *p* < 0.01. PE, preeclampsia; SD, standard deviation; IQR, interquartile range

Dividing the PE group into two subgroups depending on the development of the disease before or after 37th week, statistical significance was found comparing pregnant women who developed term PE (1.19 IQR: 0.66–1.58) with the IUGR group; *p* = 0.045 (Table 4).

**Table 4:** PAPP-A of outcome groups, insolated into preterm PE and late PE

Characteristics	Statistics	No impaired placentation disease	Preterm preeclampsia, < 37w	Late preeclampsia, ≥ 37w	Intrauterine growth restriction	Gestational hypertension
		*(n* = 360)	(*n* = 19)	(*n* = 13)	(*n* = 16)	(*n* = 14)
MoM PAPP-A	Mean (SD)	1.00 (0.50)	1.07 (0.60)	1.27 (0.68)	0.79 (0.29)	0.79 (0.65)
	Median (IQR)	0.90 (0.63–1.21)	1.04 (0.65–1.26)	1.19 (0.66–1.58) (b*)	0.83 (0.59–0.98)	0.51 (0.34–1.45)

(a) Comparison with normal, (b) comparison with intrauterine growth restriction, (c) comparison with gestational hypertension, (d) comparison with PE ≥ 37w. There was no statistical significance with (a), (c), and (d). *Significant at *p* < 0.05. **Significant at *p* < 0.01. SD, standard deviation; IQR, interquartile range

Two patients in the group of PE developed also IUGR. The value of PAPP-A in the group of PE plus IUGR was very low compared with other groups (mean 0.6 (SD 0.6); median 0.6 (IQR 0.5–0.6)).

## Discussion

Impaired placental disease is the new name that is currently used to call those pregnancy illnesses which have inappropriate placentation. For decades ago, clinical trials have been able to confirm that preeclampsia (PE), especially early-onset PE, and intrauterine growth restriction (IUGR) have a similar physiopathologic origin: the impaired placentation [1–3].

Trials about this group of obstetric diseases have focused on physiopathology, and there is enough evidence that one of the most important biomarkers for the prediction of PE and IUGR is the PAPP-A [37]; moreover, the screening combined test from the Fetal Medicine Foundation (FMF) group, which uses this gestational protein, is, nowadays, the most accurate methods of prognostication of the pregnancy at first trimester [2, 22, 24, 26, 27, 30, 38–40]. This model is also used to predict other hypertensive disorders of pregnancy, such as GH [14]. The predictive ability of this protein is also evaluated at the third trimester, but its efficiency decreases out of the first trimester of pregnancy [41].

Nearly one quarter of maternal deaths in Latin America are associated with hypertensive disorders during pregnancy, and Colombia is one of the countries with the highest incidence of PE [13]. This is the most important reason for researching this topic in pregnant Colombian women and investigating which would be the most accurate screening test to counsel preventive treatment in those women who need it [4, 5, 7, 12].

The results of this study, done in a high prevalence of preeclampsia population, also show the low level of PAPP-A in the groups which developed GH or IUGR compared to the group of patients without any impaired placentation outcomes. However, it does not occur when the PE group is analyzed. These results of pregnant Colombian women, curiously, demonstrate that the level of PAPP-A at first trimester in the sample of women who developed PE was higher than that when they only developed GH or IUGR. Most of the trials have focused on the prediction of PE and current models establish the use of PAPP-A to screen PE, finding lower levels of PAPP-A in patients who develop PE or IUGR [17, 18, 20, 22, 42]. However, some studies in other populations report similar results to this work. Saruhan et al. investigated if PAPP-A levels at first trimester were associated with adverse pregnancy outcomes, concluding that PAPP-A was not useful to prognosticate adverse outcomes [42]. Also, Ragnhild et al. reported higher PAPP-A values in the group of patients who had severe PE than those in the group of patients who only developed GH [43]. This is similar to the result of this study when the PE group is separated into patient with developing of PE before or after 34 weeks of pregnancy. Nevertheless, it is not the same when the subgroups of PE are separated using the limit of 37 weeks of pregnancy. It seems that the results of our study could be consistent with the work of Ragnhild et al.

In contrast, the patients who developed GH presented very low levels of PAPP-A. Most of the previous research had not found results like that [19, 21, 44, 45] and reported that PAPP-A is lowest in patients that developed early-onset PE and lower than that in patients who only had gestational hypertension. However, some studies found lower values of PAPP-A in patients with GH than those in the group of patients with PE like this work in Colombian pregnant women [38].

Levels of PAPP-A in this study are only similar to that in other international works about this biomarker in the outcome group of IUGR [23, 28, 37]. Surprisingly, the PAPP-A value in the group of patients who did not develop any impaired placentation disease was lower than in other studies [19, 21, 28, 43–46].

This work has used an unselected population with a high proportion of comorbidities (3.3%) and a high incidence of hypertensive disorders of pregnancy (10.9%), mainly PE (7.6%). Some ethnic origins are more prone to suffering hypertensive disorders during pregnancy, but because of that, this study employed data of MoMs to compare outcome groups, in order to standardize the population’s characteristics. Therefore, the results of PAPP-A in the PE group are unexpected, although other research groups have reported results that concluded that PAPP-A losses efficacy when it is used in populations with high comorbidity [43].

The most remarkable result of our work is the data of PAPP-A in Colombian women who developed PE, not only because it seems that there is no statistical significance compared to women who did not develop any impaired placentation disease but because the mean value of PAPP-A in these patients are upper than the mean value of patients without adverse outcomes. This could not be sustained by any of the theories about how the impair trophoblast invasion process is related to a low serum PAPP-A as in other works, where lower levels than the fifth percentile are reported to increase the risk of developing impaired placentation disease: intrauterine growth restriction (adjusted odds ratio, 2.9; 95% confidence interval [CI], 2.0–4.1) and preeclampsia (adjusted odds ratio, 2.3; 95% CI, 1.6–3.3) [17]. The findings of many studies have confirmed that the serum concentrations of PAPP-A are decreased at 11 to 14 weeks of gestation in women who develop hypertensive disorders during pregnancy, and it is more evident in those pregnant women who have early-onset PE. This study also separated women with early-onset PE and late-onset PE to review the data of the PE group, and this subanalysis could confirm that the group of early-onset PE presents the highest data of PAPP-A on this classification, although the group that presented the greatest level of PAPP-A was the term PE group. In addition, few previous papers describe the levels of PAPP-A higher than 1 MoMs, as this work reported [43]. These findings create a great doubt about the normal ranges of this biomarker in populations where PE has a higher incidence. Moreover, it raises the question if it is correct to use the normal limit of the range of PAPP-A basis on studies in European population. Further publications of the completed research will be written to be able to define why serum PAPP-A is so high in the PE group of Colombian pregnant women.

One of the most important hypotheses about the reason of this finding is related to treatment with ASA since first trimester of pregnancy. The complications of the hypertensive disorders of pregnancy can be reduced with an adequate intake of ASA in the high-risk population [24, 47]. This ongoing research study is a double-blind, and each patient was treated by their obstetrician without any commentary or recommendation before the screening realized at first trimester of pregnancy by the investigator staff. Currently, most of the patients who have high risk of PE uses ASA during pregnancy, and it is not ethical to avoid this intake. Because of that, there is the hypothesis that women who have high risk of PE perhaps did not develop it because they took ASA during pregnancy. This treatment could have changed the outcome findings, so the conclusions must be very careful when outcomes are measured; perhaps final outcomes had been more serious if this prophylactic treatment would not have ordered at the beginning of the pregnancy [48].

The main limitations of this study were that we incurred a random error because of the small sample size. Further, a selection bias was observed, given that the patients included in this study corresponded to a specific population of Bogotá. However, this work presents first findings of an ambitious research in a population where chronic illnesses related to hypertension diseases during pregnancy have a high prevalence. To study the behavior of these diseases in this type of population has huge importance because the improvement of screening and prevention strategies could decrease maternal and perinatal morbidity and mortality worldwide.

In conclusion, the results of this study demonstrate that the levels of PAPP-A at first trimester in the sample of women who developed GH and IUGR were low. Remarkably, the GH group had the lowest PAPP-A values in this sample of pregnant women. PAPP-A levels in the patients who developed PE were remarkably higher than those in most of works reported before, and this could have clinical implications that need clarification to improve the screening of hypertensive diseases during pregnancy.

## Data Availability

All data is safeguarded by the El Bosque Research Group of Maternal Fetal Medicine and Gynecology, Universidad El Bosque, Bogotá, Colombia.
